# Magnetic resonance imaging compared to ultrasonography in giant cell arteritis: a cross-sectional study

**DOI:** 10.1186/s13075-020-02335-4

**Published:** 2020-10-19

**Authors:** Ashley Yip, Elizabeth Torrey Jernberg, Mohammad Bardi, Julia Geiger, Frode Lohne, Wolfgang Andreas Schmidt, Geirmund Myklebust, Andreas P. Diamantopoulos

**Affiliations:** 1 University of British Columbia, Vancouver, BC Canada; 2 University of Washington/Virginia Mason Medical Center, Seattle, WA USA; 3 Department of Diagnostic Imaging, University Children’s Hospital Zurich, Zurich, Switzerland; 4 Department of Radiology, Hospital of Southern Norway Trust Kristiansand, Kristiansand, Norway; 5 Immanuel Krankenhaus Berlin, Medical Center for Rheumatology Berlin-Buch, Berlin, Germany; 6 Department of Rheumatology, Hospital of Southern Norway Trust Kristiansand, Kristiansand, Norway; 7 Department of Rheumatology, Martina Hansens Hospital, Bærum, Norway

**Keywords:** Vasculitis, Giant cell arteritis, Ultrasound, Magnetic resonance imaging

## Abstract

**Background:**

There has been a shift in recent years to using ultrasound (US) and magnetic resonance imaging (MRI) as first-line investigations for suspected cranial large vessel vasculitis (LVV) and is a new recommendation by the EULAR 2018 guidelines for imaging in LVV. This cross-sectional study compares the performance of US and MRI and contrast-enhanced magnetic resonance angiography (MRA) for detecting vasculitis in patients with giant cell arteritis (GCA).

**Methods:**

Patients with new-onset or already diagnosed GCA were recruited. The common temporal arteries and supra-aortic large vessels were evaluated by US and MRI/MRA. Blinded experts read the images and applied a dichotomous score (vasculitis: yes/no) in each vessel.

**Results:**

Thirty-seven patients with giant cell arteritis (GCA) were recruited. Two patients were excluded. Of the remaining patients, nine had new-onset disease and 26 had established disease. Mean age was 71 years, and median C-reactive protein (CRP) was 7.5 mg/L. The median time between US and MRI was 1 day. Overall, US revealed vasculitic changes more frequently than MRI (*p* < 0.001). US detected vascular changes in 37% of vessels compared to 21% with MRI. Among patients with chronic disease, US detected vascular changes in 23% of vessels compared to 7% with MRI in (*p* < 0.001). The same was true for patients with new-onset disease. US detected vasculitic changes in 22% of vessels and MRI detected disease in 6% (*p* = 0.0004). Compared to contrast-enhanced MRA, US was more sensitive in detecting vasculitic changes in the large arteries, including the axillary, carotid, and subclavian arteries.

**Conclusion:**

US more frequently detects vasculitic changes in the large arteries compared to contrast-enhanced MRA. When evaluating the cranial vessels, US performs similarly to MRI. This data supports the recommendation that US be considered as a first-line evaluation in patients suspected to have GCA.

## Background

Giant cell arteritis (GCA) is a type of large vessel vasculitis (LVV) that affects the walls of medium- and large-sized arteries. Granulomatous inflammation in GCA causes thickening of the arterial wall with subsequent stenosis, and in some cases, complete occlusion resulting in ischemic damage of tissue distal to the affected vessel. Some patients may develop aneurysms, especially in long-standing disease [1].

GCA affects individuals older than 50 years of age [2, 3]. The annual incidence of GCA in Norway is one of the highest worldwide, affecting 29–32 patients per 100,000 population aged 50 years or more [4, 5].

GCA may affect the cranial arteries (e.g., temporal artery) only, extracranial large arteries only, or both the cranial and extracranial arteries. LVV has been observed in up to 67% of patients with temporal artery biopsies showing GCA and up to 83% in GCA patients evaluated by positron emission tomography (PET) scan [6, 7]. The most common large vessels affected by GCA are the branches of the aortic arch, including the carotid, subclavian, and axillary arteries. The vertebral arteries and the aorta may also be involved. Stenosis, occlusions, and aneurysms are often seen in the large vessels with subsequent ischemia in vital organs including the heart, lungs, kidneys, and brain [8].

As the 1990 American College of Rheumatology (ACR) classification criteria for GCA and temporal artery biopsy primarily identifies patients with cranial involvement in GCA, vascular imaging plays an important role in the diagnosis of GCA and other forms of LVV such as TAK [9]. Ultrasound (US), magnetic resonance imaging (MRI) including magnetic resonance angiography (MRA) with contrast enhancement, computed tomography angiography (CTA), and FDG-PET/computed tomography (FDG-PET/CT) are used to evaluate vessel involvement in LVV [10, 11].

US is an inexpensive method of examining medium and large vessels and provides high-resolution images of the superficial blood vessels (temporal, carotid, vertebral, axillary, and subclavian arteries). Although the descending thoracic aorta is unable to be evaluated by transthoracic US, US can evaluate the aortic arch and the ascending aorta [12, 13]. With US, inflammation of the arterial wall is observed in B mode as homogenous enlargement of the inner two layers, the intima and media, together called the intima-media complex (IMC). When seen in a transverse view, intima-media thickening appears as a hypoechoic rim around the vessel, called a halo. Stenosis can be confirmed with visualization of arterial lumen narrowing with color Doppler or by increased flow velocity with duplex Doppler. US is readily available without long wait times or insurance appeals, and individuals can be examined in a timely manner. Because of these advantages, the European League Against Rheumatism (EULAR) recommends US as the preferred imaging modality in patients with suspected GCA [12].

MRI can confirm the diagnosis of vasculitis in superficial and deep vessels. With MRI, vascular inflammation is observed as increased vessel wall thickness and edema with increased mural enhancement on high-resolution post-contrast T1-weighted images. MRA with contrast enhancement can reveal segmental stenosis of the affected arteries. MRI may not be able to be performed in patients with relative contraindications to this imaging modality including renal failure, pregnancy, indwelling pacemaker or defibrillator, history of working with metal, or claustrophobia.

The aim of this study is to present a head-to-head comparison of the diagnostic accuracy of US and MRI of the temporal arteries and contrast-enhanced MRA of the supra-aortic large vessels in patients with GCA.

## Methods

### Patient recruitment

In this cross-sectional study, patients diagnosed with GCA were recruited between 1 January 2014 and 1 January 2015, referred from the outpatient clinic of the Department of Rheumatology, Hospital of Southern Norway Trust.

Patients were included if they met the ACR 1990 classification criteria for GCA [9]. The diagnosis was based on clinical assessment, in conjunction with imaging of temporal arteries and large supra-aortic vessels (US, MRA, CTA, or FDG-PET/CT) and/or biopsy of the temporal artery (Supplementary Table S1). Patients were re-evaluated at 6 and 12 months after study entry, and diagnosis was confirmed by clinical re-evaluation after 1 year. Patients with new-onset and long-standing GCA were recruited. New-onset disease was defined as disease onset within 2 months of study recruitment. All patients with new-onset disease received prednisolone 60 mg at the time of US examination, except for one patient, who received 20 mg. Long-standing disease was defined as disease diagnosed at least 6 months prior to study entry. Participants with chronic disease were classified as having either active or inactive disease according to clinical evaluation. Active disease was defined as the presence of typical signs or symptoms of GCA (including polymyalgia symptoms, headache, claudication in the arms or jaw, or fatigue not attributable to other causes) and an increase in inflammatory markers [14]. Remission was defined as the absence of all clinical signs and symptoms attributable to GCA and normalization of inflammatory markers [14]. Patients with moderate to severe kidney failure or known allergic reactions to contrast agents were excluded. All patients underwent US evaluation of the temporal arteries and supra-aortic large vessels. A clinical assessment and C-reactive protein (CRP) were done on the same day of US evaluation. MRI/MRA of the same vessels was performed within 1 week after US evaluation.

### Ultrasound (US) examination

**Fig. 1:**
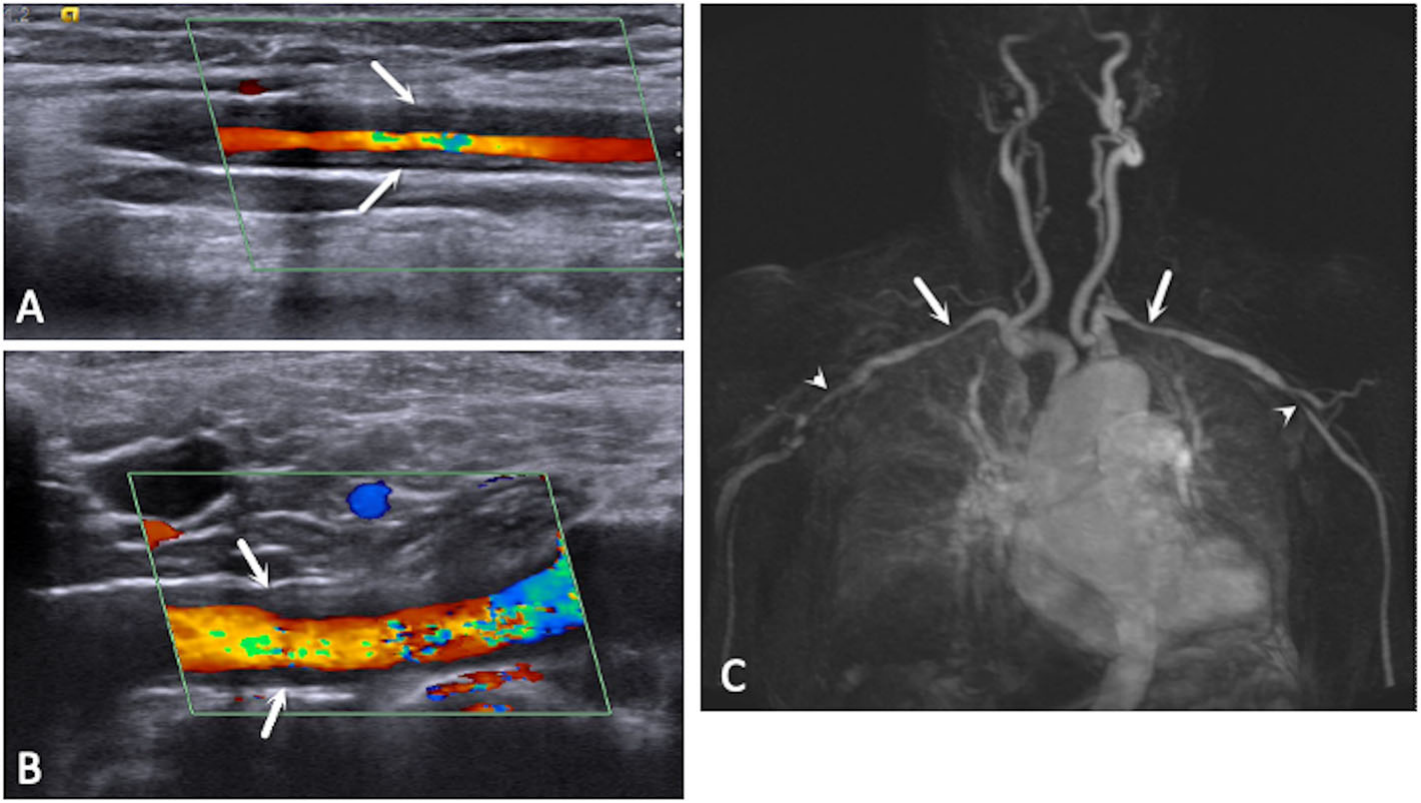
Ultrasound images revealing homogeneous hypoechoic increased intima-media thickness of the axillary (**a**) and subclavian (**b**) arteries (white arrows). Magnetic resonance angiography (MRA) (**c**) of the same patient revealing large vessel involvement, with stenosis of both subclavian arteries (white arrows) and axillary arteries (arrowheads)

All patients underwent US evaluation with a Siemens Acuson S-2000 with two multi-frequency linear transducers (6–18 MHz and 4–10 MHz) for the temporal artery and superficial supra-aortic vessels and a phased-array transducer (2–5 MHz) for the examination of the ascending aorta and aortic arch. Doppler settings were optimized according to the published guidelines for the use of Doppler US in LVV [15]. Cine clips of 3 s were recorded in both B mode and color Doppler for US. Measurement of the intima-media complex (IMC) thickness of the arteries was performed with static images. The highest IMC thickness measurement was recorded in longitudinal and transverse views. The common temporal, carotid, subclavian, vertebral, and axillary arteries; aortic arch; and ascending thoracic aorta were evaluated. Vasculitis by US was defined as a homogeneous hypoechoic thickening of the vessel with increased IMC thickness (Fig. 1a, b). Cut-off values for abnormal IMC thickness were 2.5 mm for the aorta, 1.5 mm for the carotid and subclavian arteries, and 1.0 mm for the vertebral and axillary arteries. Data in this study was collected before cut-off values for normal IMC thickness had been defined. Abnormal IMC thickness was later defined as 0.42, 0.34, 0.29, 0.37, and 1.0 mm for the common temporal, frontal temporal, parietal temporal, facial, and axillary arteries, respectively [16]. For the temporal artery, the presence of a halo (a circumferential, hypoechoic thickening of IMC in transverse and longitudinal views) was considered as a marker of vasculitis.

### Magnetic resonance imaging (MRI)

1.5-Tesla (1.5 T) MRI was performed in all patients in two sequences. High-resolution T1-weighted spin echo sequences with fat saturation were obtained to assess the cranial arteries, and contrast-enhanced MRA was used to assess the thoracic aorta and supra-aortic arteries (Supplementary Data S1) [10, 17]. Vasculitic changes on MRI are seen as mural thickening. Previously established criteria were used to define mural thickening of the cranial arteries [18, 19]. Mural thickening was evaluated using a 4-point ranking scale: 1 is no mural thickening and no mural enhancement, 2 is no mural thickening with only slight contrast enhancement, 3 is mural thickening and prominent mural enhancement, and 4 is strong mural thickening and strong mural enhancement. In MRA, information about the lumen of the artery and inflammation was assessed indirectly by stenosis (Fig. 1c). Additional transverse post-gadolinium sequences in the large vessels were not performed.

Anonymized US clips and MRI images were recorded and stored in a database at the Hospital of Southern Norway.

Locally, one ultrasonographer (APD) with 5 years of experience and one radiologist (FL) with 1 year of experience acquired and interpreted the US and MRI images, respectively. The two local evaluators were not blinded to clinical and imaging data. The anonymized images were then reviewed by two external experts, one in vascular ultrasound (WAS) and one in MRI (JG). Both external readers were blinded to previous imaging, as well as clinical and laboratory data, and unaware of the distribution and size of the vasculitic lesions. The external readers applied a dichotomous score (vasculitis: yes/no) to each evaluated vessel. In order to control for bias, interpretation by the two external experts was used to confirm the diagnosis of vasculitis. The identification of vasculitis in any vessel represented an independent result.

### Costs of imaging

The cost of the ultrasound examination was approximately 1000 Norwegian crowns (NOK) ($100 USD), while an MRI and MRA with contrast enhancement of cranial and large vessels cost 4000 NOK ($430 USD).

### Statistical analysis

Due to the lack of previous studies comparing US to MRA with contrast enhancement, the study sample was not calculated, and consequently, the study was exploratory. Interobserver agreement between the two sonographers (WAS and ADP) and the two radiologists (JG and FL) was assessed using Cohen’s kappa statistics (poor agreement: kappa < 0.40; moderate agreement: kappa 0.40–0.59; good agreement: kappa 0.60–0.79; excellent agreement: kappa ≥ 0.80). A significant difference between US and MRI was calculated using the McNemar test or the exact McNemar test when data were sparse. All analyses were performed using SAS 9.4. A Poisson regression model was performed to determine the predictors on the number of vessels with vasculitic changes on imaging. The predictors of interest were age, sex, disease status, and CRP.

## Results

Thirty-seven patients were recruited during the inclusion period. Two patients were excluded: in one patient, MRA was contraindicated due to the presence of a pacemaker, and in the second, severe back pain did not allow for MRA examination. Of the remaining 35 patients, 13 (37%) were male and 22 (63%) were female. The median age was 71 years (IQR 63–78 years). Nine patients had new-onset disease and 26 had established disease (median disease duration 2.5 years, (0–4 years)). Among the patients with new-onset disease, seven had active disease and two were in remission. Of the patients with established disease, 19 patients were in clinical remission and seven patients had active disease. The median time between US and MRI examinations was 1 day (IQR 0–2 days). The median CRP was 7.5 mg/L (IQR 3–16 mg/L). Patient characteristics are shown in Table 1.

**Table 1:** Patient characteristics

	All	New-onset GCA	Established GCA
*Gender*			
Female	22	5	17
Male	13	4	9
Mean age, years	71	72	65
Disease duration, mean in years	2.5 (range 0–4)	< 2 months	2.9 (range 0–8)
*Disease onset*			
Newly diagnosed	9		
Established disease	26		
Visual manifestations	5	0	5
Jaw claudication	1	1	0
Arm claudication	3	1	2
Patients having temporal artery biopsy (TAB)	23	2	21
TAB positive	15	1	14
CRP (range) g/L	7.5 (1–200)	24 (4–200)	4 (1–57)
Corticosteroid dose (range) mg/day	5 (0–60)	60 (20–60)	5 (0–25)
*Steroid sparing medications*			
Methotrexate	4	0	4
Leflunomide	2	0	2
Etanercept	1	0	1

*GCA* giant cell arteritis, *TAB* temporal artery biopsy, *CRP* C-reactive protein

**Fig. 2:**
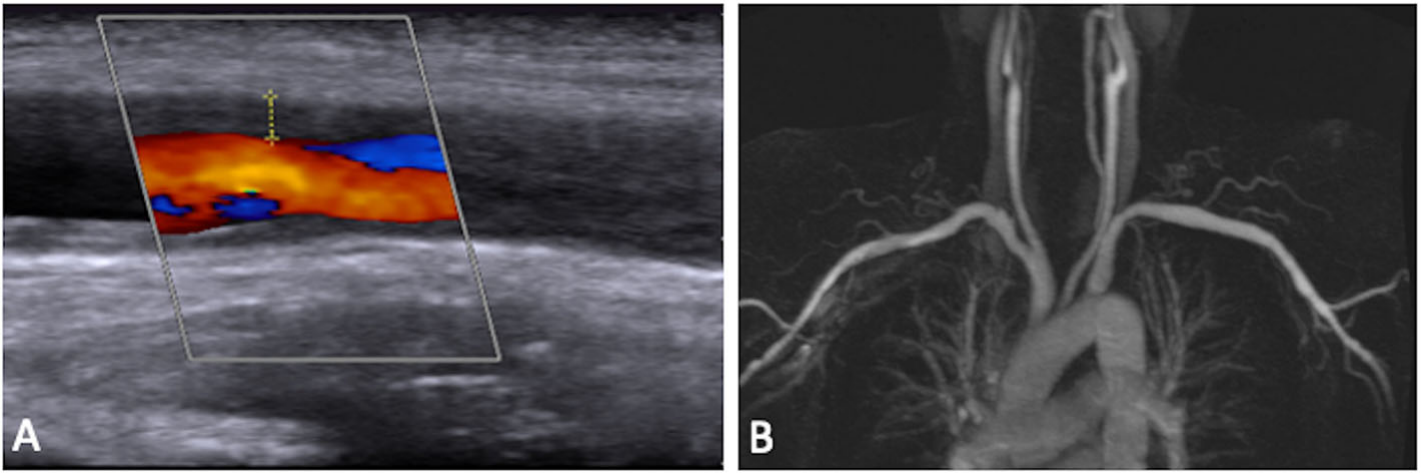
Ultrasound images (**a**) revealing increased intima-media thickness of the right axillary artery, measured at 1.8 mm on the upper vessel wall, compared to magnetic resonance angiography (**b**) in the same patient, which did not reveal evidence of vasculitis in the same vessel

The temporal arteries and the supra-aortic vessels in which US, MRI, or both modalities revealed vasculitic changes are presented in Supplemental Table S2 with *p* values presented in Supplemental Table S3. Overall, US detected vasculitic changes more frequently than MRI in the nine patients with new-onset disease, with changes reported in 77 vessels with US compared to 55 vessels with MRI (*p* = 0.0004). However, there was no statistical disagreement between US and MRI for any individual vessel. US detected disease more frequently than MRI in patients with chronic disease (*p* < 0.001). When examining individual vessels, US was more sensitive than MRA in detecting changes in the axillary (Lt *p* ≤ 0.001, Rt *p* < 0.0003), carotid (Lt *p* < 0.02, Rt *p* < 0.001), and subclavian arteries (Lt *p* < 0.0005, Rt *p* < 0.02) (Fig. 2), whereas MRI was more sensitive in detecting vasculitic changes in the right temporal frontal artery (Supplemental Table S2 and S3). For the remainder of the vessels, there was no statistical difference between the imaging modalities in the ability of each imaging modality to detect vasculitic changes. Of note, there was an excellent interobserver agreement between the ultrasonographers (kappa 0.84; 95% CI 0.79–0.89), whereas there was moderate agreement between the radiologists (kappa 0.44; 95% CI 0.33–0.54) (Table 2).

**Table 2:** Cohen’s kappa for interobserver agreement between sonographers and radiologists

Vessel	Sonographers	Radiologists
Lt axillary	0.94 (95% CI 0.83–1.00)	0.48 (95% CI − 0.12–1.00)
Lt carotid	0.68 (95% CI 0.42–0.94)	0.00 (95% CI 0.00–0.00)
Lt subclavian	1.00 (95% CI 1.00–1.00)	0.43 (95% CI − 0.03–0.90)
Lt temporal	0.94 (95% CI 0.83–1.00)	0.25 (95% CI − 0.03–0.53)
Lt frontal	0.65 (95% CI 0.40–0.90)	0.35 (95% CI 0.01–0.68)
Lt parietal	0.76 (95% CI 0.55–0.98)	0.31 (95% CI −0.03–0.65)
Lt vertebral	0.72 (95% CI 0.36–1.00)	Not calculated
Rt axillary	1.00 (95% CI 1.00–1.00)	− 0.05 (95% CI − 0.14–0.04)
Rt carotid	0.43 (95% CI 0.13–0.73)	0.65 (95% CI 0.02–1.00)
Rt subclavian	0.82 (95% CI 0.63–1.00)	0.20 (95% CI − 0.26–0.67)
Rt temporal	1.00 (95% CI 1.00–1.00)	0.44 (95% CI 0.14–0.75)
Rt frontal	0.77 (95% CI 0.52–1.00)	0.49 (95% CI 0.19–0.78)
Rt parietal	0.82 (95% CI 0.63–1.00)	0.29 (95% CI 0.02–0.57)
Rt vertebral	1.00 (95% CI 1.00–1.00)	Not calculated
Thoracic aorta	0.72 (95% CI 0.35–1.00)	0.00 (95% CI 0.00–0.00)
All vessels	0.84 (95% CI 0.79–0.89)	0.44 (95% CI 0.33–0.54)

NB. Statistics could not be calculated in the left and right vertebral arteries among the radiologists because data was too sparse

**Fig. 3:**
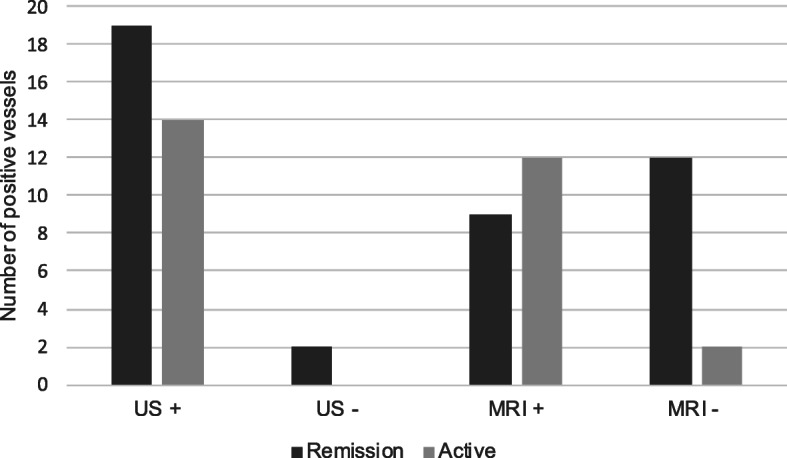
Vasculitic changes on imaging and disease activity. Normal magnetic resonance imaging (MRI) is significantly associated with disease remission (*p* = 0.01), whereas vasculitic changes may remain visible on ultrasound (US) regardless of disease activity (*p* = 0.23)

**Fig. 4:**
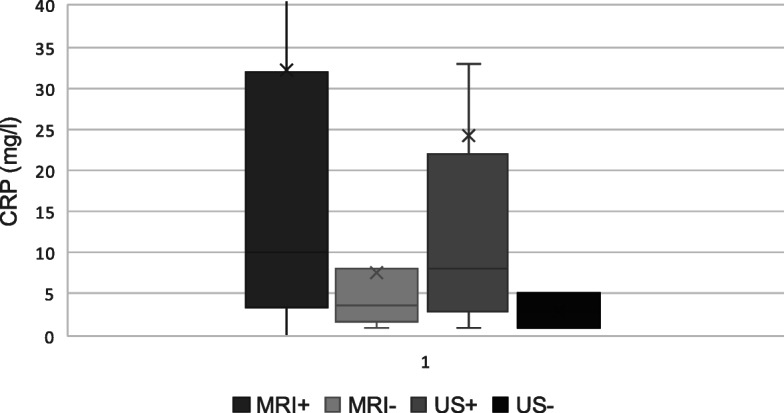
Distribution of C-reactive protein (CRP) by group. CRP was similar between the two imaging modalities. CRP was significantly correlated with vasculitic changes on MRI (*p* = 0.04), but not with US (*p* = 0.50). X represents mean CRP; middle bar represents the median CRP, and whiskers represent the upper and lower quartiles

The correlation between vasculitic changes on imaging and active disease was evaluated. The lack of vasculitic changes on MRI/MRA was significantly associated with disease remission, whereas with US, vasculitic changes were seen in both active and inactive disease. There was 45.7% concordance (*p* = 0.23) between US and disease activity, whereas there was 68.6% concordance (*p* = 0.01) for MRI (Fig. 3). Among the two imaging modalities, the average CRP was similar (Fig. 4).

Poisson regression models were used to identify predictors of the number of vessels with vasculitic changes on imaging. Active disease significantly predicted a higher number of vessels affected by MRI (*p* = 0.004), whereas CRP predicted a higher number of vasculitic changes detected by US (*p* = 0.02).

## Discussion

Historically, the diagnosis of GCA has been made on clinical grounds and confirmed with temporal artery biopsy. There has been a shift in recent years to using US and MRI as first-line investigations for suspected cranial LVV and is a new recommendation by the EULAR 2018 guidelines for imaging in LVV [12, 20, 21]. A recent meta-analysis reported US of the temporal arteries had a pooled sensitivity of 77% and specificity of 96%. High-resolution 3-T MRI had a pooled sensitivity of 77% and specificity of 88% [21]. The EULAR recommendations for imaging in LVV state a need for prospective studies directly comparing US and MRI.

The EULAR recommendations for imaging in LVV recommend the use of high-resolution 3-T MRI; however, studies comparing high-resolution 3-T MRI to US may not be applicable in practice as most centers only have access to 1-T or 1.5-T MRI machines [12, 21].

Our study showed that US was able to detect vasculitic changes similarly to 1.5-T MRI. There was no statistical difference between US and MRI in any of the cranial vessels, except the right frontal artery where MRI detected vasculitis in 17 (52%) patients and US detected changes in 7 (21%) (*p* < 0.002). Previous studies suggest 1.5-T MRI is less sensitive than US for detecting temporal artery vasculitis; however, our study did not support this finding [22].

This is the first study to compare US to MRA for the examination of extracranial large vessels in GCA. US detected vasculitic changes in the supra-aortic arteries more frequently than MRA. For example, US detected disease in the left axillary artery in 19 patients (58%) and in the right axillary artery in 25 patients (76%) compared to 3 (9%) and 5 (15%) with MRA, respectively (*p* < 0.001 and < 0.0003, respectively). This may be due to the fact that US provides higher resolution than contrast-enhanced MRA. MRA provides information about the lumen of the artery, and inflammation is assessed indirectly by stenosis. Minor vasculitic changes within the walls of the vessel may not have been detected without additional transverse post-gadolinium sequences in the large vessels. The abdominal aorta was omitted from analysis because additional MRA images would be required to evaluate the vessel in full. This may have contributed to the lower sensitivity of MRA compared to US in the large vessels. Because the axillary artery is one of the most common vessels involved in GCA, these findings suggest that US will perform better than MRA for the detection of GCA in the large arteries.

In patients with new-onset disease, glucocorticoids were initiated at the time of US examination, but for those with chronic disease, data regarding the initiation of glucocorticoids was not obtained. One study found that the sensitivity of US and MRI to detect vasculitis in the cranial vessels decreases similarly with glucocorticoid use [23]. There is marked loss of sensitivity of US to 50% and MRI to 56% after more than 4 days of glucocorticoid treatment. However, there are no studies comparing the effects of glucocorticoid use on US and MRA of the large vessels. The use of glucocorticoids may affect the sensitivity of MRA more so than US and may account for the higher detection rate with US. Furthermore, this may explain the higher concordance seen between MRA and disease activity. Resolution of contrast enhancement on MRA is reduced after 4–5 days of glucocorticoid therapy, whereas large vessel changes on US may remain detectable for months in the majority of patients, even after normalization of systemic inflammatory markers [24, 25]. The probability for MRA to detect vessel wall enhancement after more than 5 days of glucocorticoid therapy is reduced by 89.3% [26]. The lasting changes seen with US may make US the preferable imaging modality for disease detection.

US is able to detect vasculitic changes in the cranial and extracranial arteries, seen as a halo sign, a positive compression sign, stenosis, or occlusion. When extracranial arteries are examined, there is a marginal improvement in sensitivity by 2%, as seen in a study that compared examination of the temporal and axillary arteries compared to temporal artery alone [27]. However, in clinical practice, most protocols include the examination of additional cranial and large vessels. In our study, US detected vasculitic changes more frequently in the extracranial arteries than MRA, but the study was not designed to examine whether evaluating additional arteries increased the sensitivity of US for the diagnosis of GCA. Further research is needed to clarify whether the evaluation of additional vessels increases diagnostic accuracy.

Rapid diagnosis of GCA using US has resulted in a significantly reduced number of GCA patients with vision loss [28]. With US, the radiation exposure of CTA and FDG-PET/CT is avoided. Furthermore, patients who have contraindications to the use of MRI and contrast agents can be imaged with US. US is less time consuming and cheaper compared to MRI. Taking into account that US detects more abnormalities in the large vessels than MRA, and the reduced costs to perform the examination compared to MRI, one could conclude that US is more cost-effective than MRI. However, our study was not designed to examine the cost-effectiveness of the two imaging modalities and this conclusion should be interpreted with caution.

The excellent interobserver agreement among the sonographers and the moderate agreement among the radiologists could be attributed to the different experience among the readers. While both ultrasonographers performed vascular ultrasound for over 5 years, the local radiologist (FL) had limited experience on the interpretation of MRI and MRA images. This finding underscores the importance of adequate training programs for all personal performing or interpreting imaging modalities which are visualizing vascular structures.

The limitations of this study include the small sample size (*n* = 35) and cross-sectional study design, whereby treating physicians were not blinded to the patient’s clinical presentation when interpreting imaging results. This was controlled for by having external blinded reviewers interpret the images. Of note, there was no control group presented to radiologists for comparison.

Data in this study was collected before cut-off values for normal IMC thickness had been defined. Abnormal IMC thickness was later defined as 0.42, 0.34, 0.29, 0.37, and 1.0 mm for the common temporal, frontal temporal, parietal temporal, facial, and axillary arteries, respectively. In addition, compression testing on US was not performed in this study. Both could have had an impact on the accuracy of US.

## Conclusion

The results of this study provide support for the utilization of US in the detection of GCA. Although the study was not powered to compare diagnostic accuracy between US and MRI/MRA, US detected vascular changes more frequently than MRA when examining the axillary, carotid, and subclavian arteries. US has previously been shown to have comparable sensitivity and specificity to MRI but is more readily available and less expensive, making it a preferable imaging modality.

## Supplementary information

**Additional file 1: Supplemental Table S1.** Modalities used to establish the diagnosis of GCA. **Supplemental Table S2.** Percentage of patients with positive imaging by US and MRI. **Supplemental Table S3.** McNemar test comparing US and MRI. **Supplemental Data S1.** MRI protocol.

## Data Availability

The datasets used and/or analyzed during the current study are available from the corresponding author on reasonable request.
