# Supplementary material for: Magnetic resonance imaging compared to ultrasonography in giant cell arteritis: a cross-sectional study
Source: Arthritis Res Ther. 2020 Oct 19;22:247. doi: 10.1186/s13075-020-02335-4 (PMC7574248; doi:10.1186/s13075-020-02335-4)
Supplement: Supplementary file 1 — Additional file 1: Supplemental Table S1. Modalities used to establish the diagnosis of GCA. Supplemental Table S2. Percentage of patients with positive imaging by US and MRI. Supplemental Table S3. McNemar test comparing US and MRI. Supplemental Data S1. MRI protocol. [file 13075_2020_2335_MOESM1_ESM.docx]

**Supplementary**

Supplemental Table S1. Modalities used to establish the diagnosis of GCA

|  | Number of patients | Percent of total patients |
| --- | --- | --- |
| Biopsy proven | 5 | 14.3% |
| US proven | 13 | 37.1% |
| Biopsy and US proven | 12 | 34.3% |
| Other imaging modalities (CTA and PET-CT) | 5 | 14.3% |
| Total | 35 | 100% |

Supplemental Table S2. Percentage of patients with positive imaging by US and MRI

| **Vessel** | **New Disease** | | | | **Chronic Disease** | | | **All Disease** | | | | |
| --- | --- | --- | --- | --- | --- | --- | --- | --- | --- | --- | --- | --- |
|  | **US Only** | **MRI Only** | **US and MRI** | **US Only** | | **MRI Only** | **US and MRI** | | **US Only** | **MRI Only** | **US and MRI** |  |
| Lt axillary | 5 of 9 (56%) | 0 of 9 (0%) | 1 of 9 (11%) | 11 of 24 (46%) | | 0 of 24 (0%) | 2 of 24 (8%) | | 16 of 33 (49%) | 0 of 33 (0%) | 3 of 33 (9%) |  |
| Lt carotid | 3 of 9 (33%) | 1 of 9 (11%) | 0 of 9 (0%) | 9 of 24 (38%) | | 1 of 24 (4%) | 0 of 24 (0%) | | 12 of 33 (36%) | 2 of 33 (6%) | 0 of 33 (0%) |  |
| Lt subclavian | 3 of 9 (33%) | 0 of 9 (0%) | 1 of 9 (11%) | 12 of 23 (52%) | | 0 of 23 (0%) | 3 of 23 (13%) | | 15 of 32 (47%) | 1 of 32 (3%) | 4 of 32 (13%) |  |
| Lt temporal | 0 of 9 (0%) | 0 of 9 (0%) | 9 of 9 (100%) | 2 of 24 (8%) | | 2 of 24 (8%) | 4 of 24 (17%) | | 2 of 33 (6%) | 2 of 33 (6%) | 13 of 33 (39%) |  |
| Lt frontal | 2 of 9 (22%) | 1 of 9 (11%) | 6 of 9 (67%) | 1 of 24 (4%) | | 3 of 24 (13%) | 2 of 24 (8%) | | 3 of 33 (9%) | 4 of 33 (12%) | 8 of 33 (24%) |  |
| Lt parietal | 0 of 9 (0%) | 1 of 9 (11%) | 7 of 9 (78%) | 6 of 24 (25%) | | 1 of 24 (4%) | 1 of 24 (4%) | | 6 of 33 (18%) | 2 of 33 (6%) | 8 of 33 (24%) |  |
| Lt vertebral | 2 of 9 (22%) | 0 of 9 (0%) | 0 of 9 (0%) | 1 of 23 (4%) | | 0 of 23 (0%) | 0 of 23 (0%) | | 3 of 32 (9%) | 0 of 32 (0%) | 0 of 32 (0%) |  |
| Rt axillary | 5 of 9 (56%) | 0 of 9 (0%) | 1 of 9 (11%) | 16 of 24 (67%) | | 1 of 24 (4%) | 3 of 24 (13%) | | 21 of 33 (64%) | 1 of 33 (3%) | 4 of 33 (12%) |  |
| Rt carotid | 2 of 9 (22%) | 1 of 9 (11%) | 0 of 9 (0%) | 11 of 24 (46%) | | 0 of 24 (0%) | 1 of 24 (4%) | | 13 of 33 (39%) | 1 of 33 (3%) | 1 of 33 (3%) |  |
| Rt subclavian | 3 of 9 (33%) | 0 of 9 (0%) | 1 of 9 (11%) | 7 of 24 (29%) | | 0 of 24 (0%) | 3 of 24 (13%) | | 10 of 33 (30%) | 0 of 33 (0%) | 4 of 33 (12%) |  |
| Rt temporal | 2 of 9 (22%) | 1 of 9 (11%) | 6 of 9 (67%) | 1 of 24 (4%) | | 5 of 24 (21%) | 3 of 24 (13%) | | 3 of 33 (9%) | 6 of 33 (18%) | 9 of 33 (27%) |  |
| Rt frontal | 0 of 9 (0%) | 2 of 9 (22%) | 7 of 9 (78%) | 0 of 24 (0%) | | 8 of 24 (33%) | 0 of 24 (0%) | | 0 of 33 (0%) | 10 of 33 (30%) | 7 of 33 (21%) |  |
| Rt parietal | 1 of 9 (11%) | 1 of 9 (11%) | 7 of 9 (78%) | 4 of 24 (17%) | | 3 of 24 (13%) | 2 of 24 (8%) | | 5 of 33 (15%) | 4 of 33 (12%) | 9 of 33 (27%) |  |
| Rt vertebral | 1 of 9 (11%) | 0 of 9 (0%) | 0 of 9 (0%) | 1 of 23 (4%) | | 0 of 23 (0%) | 0 of 23 (0%) | | 2 of 32 (6%) | 0 of 32 (0%) | 0 of 32 (0%) |  |
| Thoracic aorta | 1 of 7 (14%) | 0 of 7 (0%) | 1 of 7 (14%) | 2 of 23 (9%) | | 0 of 23 (0%) | 0 of 23 (0%) | | 3 of 30 (10%) | 0 of 30 (0%) | 1 of 30 (3%) |  |
| All vessels | 30 of 136 (22%) | 8 of 136 (6%) | 47 of 136 (35%) | 84 of 365 (23%) | | 24 of 365 (7%) | 24 of 365 (7%) | | 114 of 501 (23%) | 32 of 501 (6%) | 71 of 501 (15%) |  |

Supplemental Table S3. McNemar test comparing US and MRI.

| **Vessel** | **New Disease** | **Chronic Disease** | **All Disease** |
| --- | --- | --- | --- |
| Lt axillary | p 0.06 | p 0.001 | p < 0.001 |
| Lt carotid | p 0.63 | p 0.01 | p < 0.01 |
| Lt subclavian | p 0.25 | p 0.0005 | p < 0.0001 |
| Lt temporal | Not calculated | p 1.00 | p 1.00 |
| Lt frontal | p 1.00 | p 0.63 | p 1.00 |
| Lt parietal | p 1.00 | p 0.13 | p 0.29 |
| Lt vertebral | p 0.50 | p 1.00 | p 0.25 |
| Rt axillary | p 0.06 | p 0.0003 | p < 0.001 |
| Rt carotid | p 1.00 | p 0.001 | p 0.002 |
| Rt subclavian | p 0.25 | p 0.02 | p 0.002 |
| Rt temporal | p 1 | p 0.22 | p 0.51 |
| Rt frontal | p 0.50 | p 0.01 | p 0.002 |
| Rt parietal | p 1 | p 1.00 | p 1.00 |
| Rt vertebral | p 1.00 | p 1.00 | p 0.50 |
| Thoracic aorta | p 1.00 | p 0.50 | p 0.25 |
| All vessels | Asymptotic p 0.0004 | Asymptotic p <.0001 | Asymptotic p < 0.001 |

NB. Exact p is reported unless otherwise stated. No agree statistics could be computed for the left temporal artery among patients with new disease, as there was complete agreement between the imaging modalities.

Supplemental Data S1: MRI protocol.

 Siemens Aera 1.5 Tesla. Cor angio (thoracic aorta + pre cerebral arteries)

- 3d Flash twist
- Section thickness 2 mm
- TE 0.99
- TR 2.62
- Pixel BW 625
- Acquisition Matrix 0\320\192\0
- Interpolar resolution 384 x 640
- Flip angle 8
- Time resolution 11,73 sec x 9 phases
- FOV 400 cm
- Tra T1 fs temporal
- T1 SE fat saturation
- Section thickness 2mm x 26 snitt
- TR 661
- TE 21
- Pixel BW 65
- Acquisition Matrix 0\512\3814\0
- Interpolar resolution 1024 x 768
- Flip angle 90
- FOV 200 cm
